# Upscaling the Use of Mixed Recycled Aggregates in Non-Structural Low Cement Concrete

**DOI:** 10.3390/ma9020091

**Published:** 2016-02-02

**Authors:** Antonio López-Uceda, Jesús Ayuso, José Ramón Jiménez, Francisco Agrela, Auxiliadora Barbudo, Jorge De Brito

**Affiliations:** 1 Construction Engineering, University of Córdoba, Ed. Leonardo Da Vinci, Campus of Rabanales, Córdoba 14071, Spain; p62louca@uco.es (A.L.-U.); ir1jiroj@uco.es (J.R.J.); ir1agsaf@uco.es (F.A.); g82bamum@uco.es (A.B.); 2 CERIS/ICIST, DECivil, Instituto Superior Técnico, Universidade de Lisboa, Av. Rovisco Pais, Lisbon 1049-001, Portugal; jb@civil.ist.utl.pt

**Keywords:** upscaled experimental study, non-structural concrete, long term mechanical performance, concrete cores, ready-mix plant, low cement content, mixed recycled aggregates

## Abstract

This research aims to produce non-structural concrete with mixed recycled aggregates (MRA) in upscaled applications with low-cement content. Four slabs were executed with concrete made with different ratios of coarse MRA (0%, 20%, 40% and 100%), using the mix design, the mixing procedures and the facilities from a nearby concrete production plant. The analysis of the long-term compressive and splitting tensile strengths in concrete cores, extracted from the slabs, allowed the highlighting of the long-term high strength development potential of MRA incorporation. The study of cast specimens produced *in situ* under the same conditions as the slabs showed, firstly, that the use of MRA has a great influence on the properties related to durability, secondly, that the loss of compressive strength for total MRA incorporation relative to control concrete increases proportionally with the class strength, and, thirdly, that the mechanical properties (including Schmidt hammer results) from the concrete slabs showed no significant differences relative to the control concrete for coarse aggregates replacements up to 40%. Therefore, this upscaled experimental study supports the application of concrete with 100% coarse MRA incorporation and low cement content in non-structural civil works such as bike lanes, gutters, ground slabs, leveling surfaces, and subgrades for foundations. To the best of the authors’ knowledge, there have not been any upscaled applications of concrete with MRA and low cement content.

## 1. Introduction

Given the amount of construction and demolition waste (CDW), approximately 750 million tonnes per year, according to the European Commission, CDW has been recently upgraded to a priority waste stream status in the European Union (EU) [1] in order to reach 70% by weight in re-use, recycling and other recovery operations by 2020, according to Directive 2008/98/CE [2], established at the European level. In a recent study in Spain, Rodríguez-Robles *et al.* [3] concluded that there are no reliable regional data on yearly generated CDW, and the most recent reliable figure in 2010 is 23 million.

CDW comes from total or partial construction or demolition of buildings and civil infrastructures. Its composition comprises numerous materials: concrete, natural aggregates, bricks, and, to a lesser extent gypsum, wood, glass, metals, and plastics among others. The two major recycled aggregates (RA) from CDW are recycled concrete aggregate (RCA), which are produced by crushing concrete, and mixed recycled aggregate (MRA), which contains an significant percentage of masonry rubble. In Southern European countries, many architectural interior building elements are ceramic. In Spain, MRA represents over 70% of total CDW aggregates [4]. Hence, Agrela *et al.* [5] established a classification for RA depending on the content of ceramic and concrete particles: If RA’s concrete content ≥90%, it is called concrete recycled aggregate; if its ceramic content is between 10% and 30%, it is named mixed recycled aggregate; finally, if its ceramic content is >30%, it is called ceramic recycled aggregate. Additionally, Silva *et al.* [6] suggested a different RA classification based on the oven-dried density, water absorption and LA abrasion value. Nowadays, the most common application of MRA in Spain is in unpaved rural roads with low daily heavy traffic, with low value added [7,8]. Additionally, Vegas *et al.* [9] mentioned that MRA have been used so far mostly in applications with low added value.

According to the Spanish Code on Structural Concrete (EHE-08) [10], a minimum strength class of 15 MPa is required for non-structural concrete, but the minimum cement content shall be 150 kg/m^3^. MRA is not allowed in any case, but RCA can be used in non-structural concrete up to 100% and up to 20% in structural concrete, in the coarse fraction in both cases. EN 206-1 requires a minimum cement content of 240 kg/m^3^ for structural concrete and 150 kg/m^3^ for non-structural concrete [11]. Standards of countries like Germany, United Kingdom, Netherlands, and Portugal allow the use of MRA in non-structural concrete [12].

In Spain, the incorporation of MRA in concrete could be an environmental-friendly value-added solution for this type of RA. To the best of the authors’ knowledge, few authors have studied MRA from CDW recycling plants, as total or partial replacement of the coarse aggregate fraction in the production of concrete [13,14,15,16,17,18]. In these studies, the minimum cement content was 240 kg/m^3^.

Martinez-Lage *et al.* [13] found that the decline in density, compressive strength and modulus of elasticity was approximately linear with the replacement ratio, and it amounted to 7%, 20%–30% and 30%–40%, respectively, in concrete containing 100% recycled aggregate.

Mas *et al.* [14] concluded that a decrease in concrete’s compressive and tensile strength takes place as the MRA ratio increases. The relative loss of strength was higher as concrete strength increased. The loss in long-term (90 days) strength, relative to the reference concrete, is less than that in the short term. MRA incorporation up to 20%–25% leads to strength decreases of less than 15%. In relation to durability, MRA mixes’ water under pressure penetration showed a linear increase with the replacement level. A long-term experimental campaign on concrete made with MRA was also suggested.

Mas *et al.* [15] analyzed the influence of the type of cement, concluding that concrete made with cement with fly ash showed a lower decrease in strength and permeability as the MRA ratio increased.

Medina *et al.* [16] found that the saturated density and mechanical performance of aggregate concrete are moderately lower than those of the reference concrete, particularly at higher RA incorporation ratios and with impurities. MRA incorporation levels up to 25% have no effect on the sorptivity of concrete. Medina *et al.* [17] found that the coarse aggregate/paste interface varied depending on the components: Mixes with inorganic materials (gravel, concrete waste and clay-based materials) exhibited a narrower and more compact interface than mixes with organic constituents (asphalt and floating particles). Rodríguez-Robles *et al.* [18] found that there was a greater negative impact on the mechanical properties of recycled aggregate concrete than found by other authors because of the high cement content they used, confirming the results of Mas *et al.* [15]. Almost all of the authors agree on the use of admixtures in order to balance the loss of workability caused by the incorporation of high-absorption MRA. Additionally, the use of MRA from CDW in concrete was suggested as a feasible option to improve the construction sector’s sustainability, as Bravo *et al.* [19,20] demonstrated.

Kou *et al.* [21] studied the influence on different properties of concrete mixes of different recycled aggregates sorted from a Hong Kong’s CDW treatment plant. Coarse RA incorporation caused a reduction in ultrasonic pulse velocity (UPV) and in compressive strength, but the quality of the different recycled aggregates did not show significant influence on both of them.

Zaharieva *et al.* [22] obtained an increase of 75% in porosity and a decrease of 3.15% in density, comparing concrete mixes of full coarse replacement of natural aggregates (NA) by MRA from a CDW treatment plant respect to control concrete made with NA.

Sheen *et al.* [23] evaluated the effect of RA from the earthquake of Chi-Chi in Taiwan on concrete. Compressive strength reduction was found in concrete made with RA relative to control concrete affected by the brick and tile content of the RA. A higher compressive strength development over the long term was also observed in unwashed RA concrete mixes than in washed ones.

Literature on MRA in real applications has not been found, with the exception of one paper by Etxeberría *et al.* [24], unlike in the case of RCA. In Shanghai, China, almost 400 m^3^ of concrete with RCA were used in an ecological green building, mostly in walls and foundations [25]. In Hong Kong, Wetland Park consumed approximately 14,300 m^3^ of concrete made with RCA, namely in ground slabs, external works, mass concrete and minor concrete works. Zhang *et al.* [26] studied the performance of RCA in beams, proving the feasibility in structural concrete of this type of recycled aggregate. Soares *et al.* [27], by means of the execution of four full-scale two-storey reinforced concrete structures, concluded that RCA from the precasting industry is suitable for incorporation in structural concrete. In Spain, Rodríguez-Robles *et al.* [28] listed some pilot projects using RCA in concrete such as sub-bases and ripraps located in the Olympic Village in Barcelona, a cable-stayed bridge in Valencia and a footbridge in Barcelona.

This study continues the research of Uceda *et al.* [29] with the aim of producing non-structural concrete with MRA, sourced directly from a CDW recycling plant in Córdoba (Spain), and low cement content (200 kg/m^3^). To the best of the authors’ knowledge, there are no upscaled applications of concrete with MRA and low cement content. This material may be applied in bike lanes, gutters, ground slabs, leveling surfaces, subgrades for foundations and similar civil works. Hence, on-site concrete slabs were executed with different ratios of coarse mixed recycled aggregate (0%, 20%, 40% and 100%), using the mix design, the mixing procedures and the facilities from a nearby concrete production plant. Long-term compressive and tensile strength were performed in cores extracted from the slabs. Schmidt hammer tests were performed on the concrete slabs. Furthermore, mechanical and durability-related properties were studied in laboratory conditions (in terms of curing), using specimens cast *in situ* under the same conditions as the slabs.

## 2. Materials and Experimental Details

### 2.1. Materials

**Figure 1:**
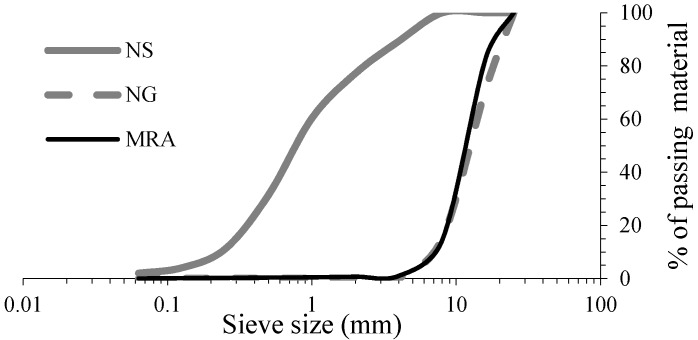
Grain size distribution of aggregates used.

Natural siliceous sand (NS), with a maximum size of 4 mm, and Portland cement-type CEM II/A-V 42.5 R, with a specific gravity of 2.89 g/cm^3^, were used in all the mixes. Two types of coarse aggregate were used: a siliceous gravel (NG) with a size range of (6–25 mm) and a mixed recycled aggregate (MRA) from a recycling plant of CDW located in Cordoba (South of Spain), which was used exactly as it came out of the plant. Figure 1 shows the grain size distribution of the aggregates used, where both coarse aggregates display similar curves. Table 1 shows the physical and chemical properties of the aggregates. A plasticizer (Conplast MR 260) and a superplasticizer (Conplast SP 420) were used simultaneously to reduce the water content and increase workability.

**Table 1:** Physical and chemical properties of the aggregates.

**Physical Properties**	**Test Standard**	**NS**	**NG**	**MRA**	**EHE-08 Requirements**
Water absorption (%)	UNE-EN 1097-6:2014 [30]	0.92	0.73	9.0	<5% General <7% RCA
Oven-dried density (g/cm^3^)	UNE-EN 1097-6:2014 [30]	2.64	2.68	2.08	-
SSD density (g/cm^3^)	UNE-EN 1097-6:2014 [30]	2.66	2.70	2.27	-
Flakiness index (%)	UNE-EN 933-3:2012 [31]	-	20.6	14.7	<35
Friability test	UNE-EN 1097-2:2010 [32]	12.4	-	-	-
Los Angeles abrasion test	UNE-EN 1367-2:2010 [33]	-	18.1	32.3	<40
Freeze-thaw resistance (%)	UNE-EN 1097-6:2014 [34]	-	-	14.0	<18%
**Chemical properties**	**Test Standard**	**NS**	**NG**	**MRA**	**EHE-08 Requirements**
Total sulphur content (% S)	UNE-EN 1744-1-11:2010 [35]	0.17	0.2	0.96	<1
Acid-soluble sulphates (% SO_3_)	UNE-EN 1744-1-12:2010 [36]	0.36	0.56	0.62	<0.8
Chlorides (%)	UNE-EN 1744-1-7:2010 [37]	<0.01	<0.01	<0.01	<0.05

The only test result that did not comply with EHE-08 [10] requirements is the water absorption of MRA. However, according to RILEM [38], this MRA could be classified as Type II aggregates, fit to be used in an up to C50/60 strength class.

Some other Spanish researches were analyzed in order to compare the material used in this study with other of the same geographical area. Rodriguez-Robles *et al.* [28] studied thirteen samples of MRA with different ceramic contents from several Spanish CDW recycling plants. The ceramic constituents’ mean value was 32%, ranging between 16.51% and 64.75%. Vegas *et al.* [9] analyzed ten different MRA produced in three recycling plants in the Basque Country, North of Spain. The ceramic material mean value was 27.4%, with a range between 12% and 43%. According to Agrela *et al.* [5], the average ceramic content of 27 MRA samples from 13 CDW recycling plants in Spain was 24%, and it ranged between 12.7% and 53.9%. Thus, in spite of the heterogeneous nature of this type of RA, the RA studied in our research is representative in terms of its ceramic composition (Table 2). Sheen *et al.* [23] used two MRA with brick and tile contents of 32% and 24%, available in Taiwan.

**Table 2:** MRA composition according to UNE-EN 933-11:2009 [39].

Components	Percentage
Asphalt	0.5
Ceramics	30.2
Mortar and concrete	44.6
Unbound aggregates	24.0
Gypsum	0.5
Others (wood, glass, plastic and metal)	0.2

### 2.2. Mix Design and Procedures

The composition of the mixes (Table 3) and the ready-mixed concrete were supplied by a ready-mix plant in Cordoba, Spain. Constant cement content (200 kg/m^3^) and water/cement ratio (0.65) were used in all mixes. Four concrete slabs were manufactured, one with natural aggregates (CC), and one for each MRA incorporation ratio: 20%, 40% and 100% (in volume), named RC20, RC40 and RC100, respectively. The slabs dimensions were 3.5 × 3.5 × 0.25 m.

**Table 3:** Composition of the concrete mixes (kg/m^3^).

Samples	Cement	Water	NS	NG	MRA	Plasticizer	Superplasticizer	Slump (cm)
CC	200	130	1070	950	0	2.13	2.39	17
RC20	200	130	1070	817	144	2.13	2.39	14
RC40	200	130	1070	613	288	2.13	2.39	12
RC100	200	130	1070	0	720	2.13	2.39	16

Two admixtures were used in all the mixes, at 9 mL/kg and 10 mL/kg of cement. The plasticizer and superplasticizer were added sequentially in order to have a slump value of 15 ± 3 cm according to UNE-EN-206-1:2008 [40]. Oliveira and Vazquez [41] obtained better results with semi-saturated RA (saturation degree of 85%–90%) than with air-dried or saturated RA. Partially saturated RA has been used in several studies [42,43,44,45,46,47]. Therefore, the MRA was watered before concrete mixing. To produce the mixes in the ready-mix plant, coarse aggregate and NS were fed into an actual truck mixer, after which the cement and 80% of the water were added sequentially and mixed for two minutes before adding the admixtures with 10% of the water for each one of them.

### 2.3. Specimens, Curing and Test Methods

**Figure 2:**
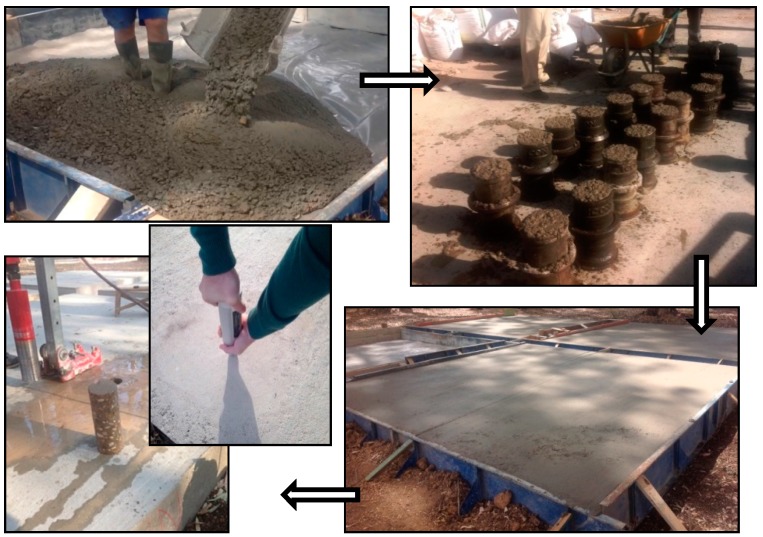
Slab execution, specimens cast and core extraction and non-destructive test.

Cylindrical specimens, Ø 150 × 300 mm, were cast during the slabs execution (Figure 2): After 24 h, specimens were demolded and stored in a wet chamber (at 23 ± 2 °C and 95% ± 5% relative humidity). Before casting the specimens, a workability test was performed for each slab (measured with the Abrams cone) (results in Table 3) according to UNE-EN 12350-2:2009 [48].

Table 4 lists the tests conducted on the cast specimens and their curing time. The value presented for each test and curing time is the average of three replicates.

A non-destructive *in situ* test, using the Schmidt hammer, was carried out at 7, 28 and 90 days, following standard UNE-EN 12504-2:2013 [49]. Five measurements were made per slab, each one corresponding to the median of ten readings. Six Ø 100 × 200 mm cylindrical concrete cores were extracted from each slab at 7, 28, 90, 180 and 365 days, in accordance with UNE EN 12504-1: 2009 [50]. Once extracted, three were tested for compressive strength and three for splitting tensile strength.

**Table 4:** Tests conducted in cast specimens.

Tests (Curing Time in Days)	Standards
Compressive strength (7, 28, 90 and 180)	UNE-EN 12390-3:2009 [51]
Splitting tensile strength (7, 28 and 90)	UNE-EN 12390-6:2009 [52]
Modulus of elasticity in compression (28)	UNE 83316:1996 [53]
Ultrasonic pulse velocity (7, 28, 90 and 180)	UNE 12504-4:2006 [54]
Density of hardened concrete (28)	UNE-EN 12390-7:2009 [55]
Porosity of hardened concrete (28)	UNE-EN 12390-7:2009 [55]
Penetration depth of water under pressure (28)	UNE-EN 12390-8:2009 [56]
Sorptivity (28)	UNE- EN 1925:1999 [57]

## 3. Results and Discussion

### 3.1. Compressive Strength

#### 3.1.1. Cast Specimens

**Figure 3:**
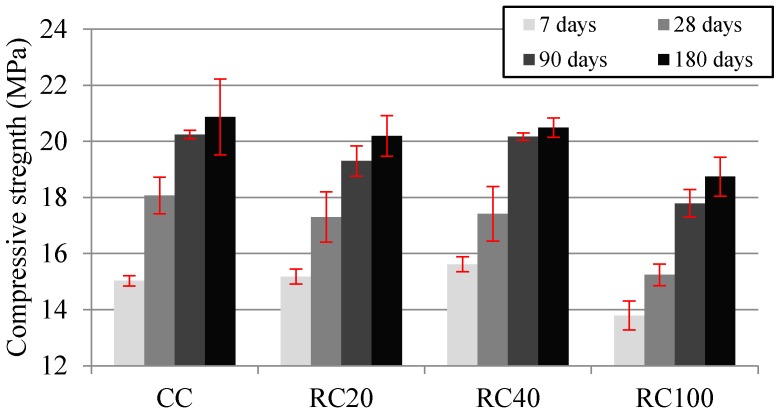
Compressive strength in cast specimens at 7, 28, 90 and 180 days.

Figure 3 shows the average compressive strength at 7, 28, 90 and 180 days and the corresponding standard deviation. The average values of the mixes with 20% and 40% of MRA incorporation ratio are very similar to those of CC at the same age except those of RC100, whose compressive strength losses relative to CC decreased over the long term: 15.7%, 12.1% and 10.2%, at 28, 90 and 180 days respectively, in agreement with Sheen *et al.* [23] but at earlier ages. This higher strength gain relative to CC over the long term may be due to the improvement of the microstructure of the interfacial transition zone (ITZ) and the increase of the bond strength between the new cement paste and MRA constituents after continuous hydration because of the presence of the mortar and concrete in the MRA used [58]. It was found that RC40 reached slightly higher compressive strength than RC20, which could be attributed to the higher RC40’s slump. Nonetheless, the differences were minor (the average at the four ages of RC40 was higher than that of RC20 by 2.38%, with differences ranging from 0.7% to 4.5%). Mas *et al.* [14] obtained a compressive strength decrease of 13% for both 20% and 40% MRA incorporation ratio at 28 days, but with more cement content. Bravo *et al.* [20], with RA with similar ceramic content and 350 kg/m^3^ of cement, obtained a ratio equal to 68.5% between the compressive strengths of the mix with total replacement at 7 and 28 days, while in our study the same ratio of the equivalent mix (RC100) was 76.3%.

**Figure 4:**
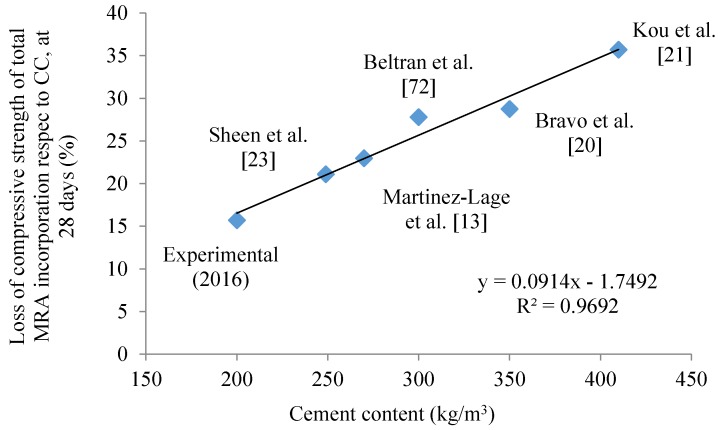
Compressive strength in cast specimens at 28 days obtained by several authors in concrete with full MRA replacement.

Figure 4 shows the loss of compressive strength of total MRA incorporation relative to reference concrete and cement content at 28 days by several authors. Mas *et al.* [15] concluded that the loss of strength is proportionally higher as concrete strength increases. In Figure 4, the idea that the loss of compressive strength in total MRA incorporation relative to control concrete is proportionally higher as cement content increases (class strength) is reinforced. This could be attributed to the fact that the higher the strength class, the higher the influence of the aggregate used on the concrete’s compressive strength is.

**Figure 5:**
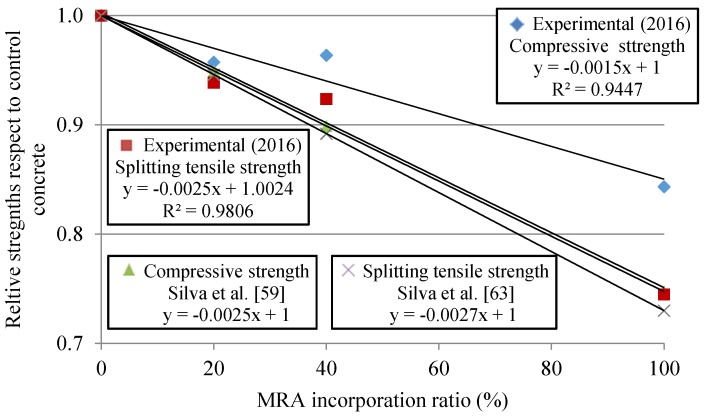
Relative compressive and splitting tensile strength in cast specimens at 28 days obtained by Silva *et al.*’s review in concrete with MRA.

Silva *et al.* [59] studied, through an extensive literature meta-analysis, the influence of RA on the compressive strength. Figure 5 shows that Silva *et al.*’s compressive strength trend reduces the concrete’s strength to a greater extent than in our study. As mentioned before, publications were found with lower cement content than the one used here; therefore, this supports the idea that low strength class leads to lower loss of the compressive strength relative to control concrete.

#### 3.1.2. Core Concrete

**Figure 6:**
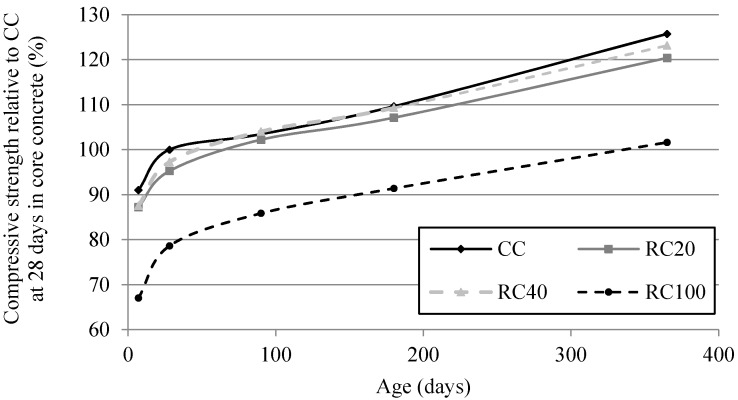
Compressive strength in core concrete relative to that of CC at 28 days.

As in cast specimens, compressive strength average values of the mixes with 20% and 40% of MRA are very similar to those of CC (15.8 MPa). As seen in Figure 6, RC100’s compressive strength took one year to reach CC’s at 28 days, as in Kou and Poon [60], but with RCA and higher cement and fly ash content.

**Figure 7:**
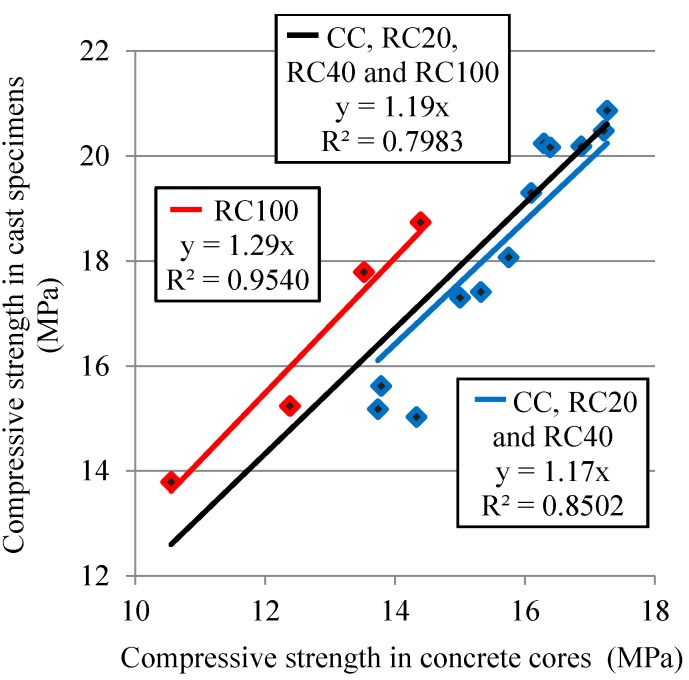
Correlation between compressive strength of concrete cores and cast specimens.

Figure 7 shows different correlations between compressive strength obtained in cast specimens and concrete core for all ages studied. By separating RC100 values, a better correlations index was found than with all the mixes (*R*^2^ = 0.7983), 0.8502 with CC, RC20 and RC40 mixes and 0.954 with RC100 mix. The higher ratio between compressive strength in cast specimens and concrete cores in RC100 (1.29) than that of up to 40% MRA incorporation (1.17) can be attributed to the fact that damage from drilling increases for poor-quality concrete [61].

### 3.2. Splitting Tensile Strength

#### 3.2.1. Cast Specimens

**Figure 8:**
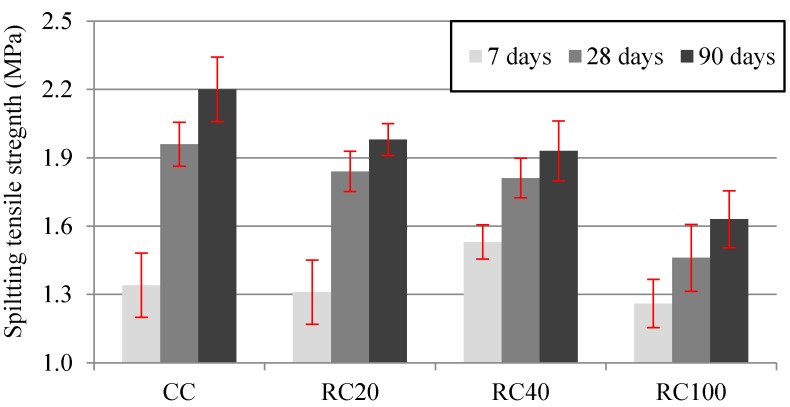
Splitting tensile strength in cast specimens at 7, 28 and 90 days.

Figure 8 shows the average splitting tensile strength at 7, 28 and 90 days and the corresponding standard deviation. At 7 days, the strength variations registered were 0.2%, −1.26% and 0.53% for RC20, RC40 and RC100 mixes respectively, relative to CC mix. There was a decrease of 25.5% relative to control concrete with full replacement after 28 days, whereas Bravo *et al.* [20] found over 30%, and Kou *et al.* [58] found a 36% loss of splitting tensile strength with 100% full MRA incorporation.

The splitting tensile strength decreased as the replacement ratio increased (Figure 5). This trend regarding MRA incorporation ratio was in agreement with Silva *et al.*’s [62] study, derived from the results of nearly 50 concrete mixes with different coarse substitution ratios of NA with MRA.

#### 3.2.2. Core Concrete

**Figure 9:**
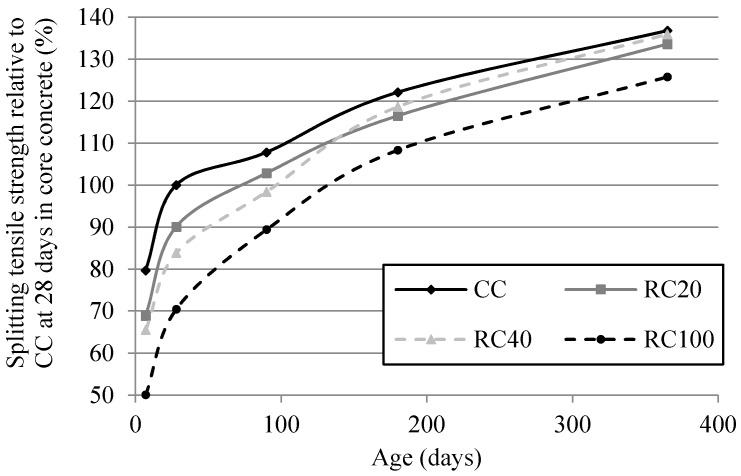
Splitting tensile strength in core concrete relative to that of CC at 28 days.

Figure 9 shows the splitting tensile strength relative to CC at 28 days. It shows that the average values of the mixes with 20% and 40% of MRA are very similar to those of CC (1.28), as for compressive strength. RC100’s splitting tensile strength took less than 180 days to reach that of the control concrete at 28 days. Kou and Poon [60] observed that, after one year, concrete with full RCA replacement had higher strength than that of the corresponding control concrete. This increasing long-term trend may be attributed to an improvement of the interstitial transition zone’s microstructure between coarse RCA and new cement paste [27]. There was a lower difference relative to control concrete over the long term in mixes with MRA incorporation. Not only does MRA induce the same effect, but it also improves it. At 7, 28, 90, 180 and 365 days, RC100’s splitting tensile strength relative to CC was 37.2%, 29.5%, 17%, 11.3% and 8.1%, respectively.

### 3.3. Modulus of Elasticity

**Figure 10:**
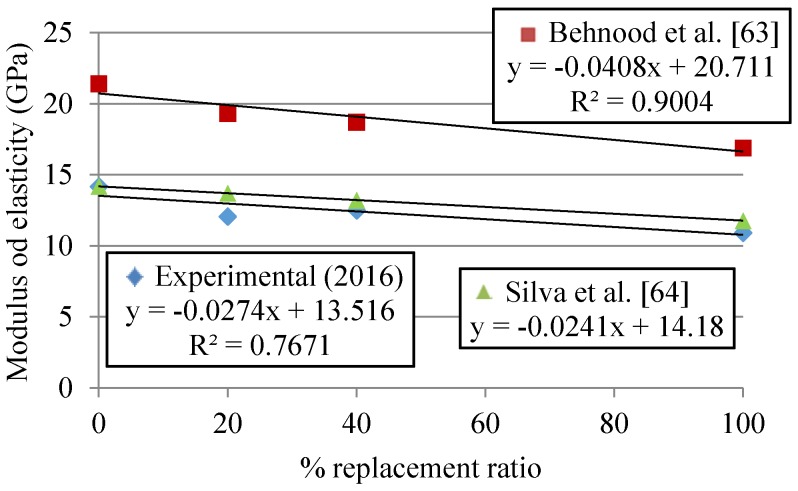
Modulus of elasticity in cast specimens with MRA incorporation ratio at 28 days.

The modulus of elasticity decreases as the MRA replacement ratio increases (Figure 10), similarly to compressive and splitting tensile strengths. Sheen *et al.* [23] obtained a higher loss of modulus of elasticity (27%, whereas in our research 23% was obtained) for full coarse replacement of NA, with similar MRA and higher cement (249 kg/m^3^ of cement). Behnood *et al.* [63] established, through extensive data collection, a model for the prediction of the modulus of elasticity according to several factors such as compressive strength, SSD density, water absorption, water-cement, coarse aggregate-cement and fine aggregate-total aggregate ratio. Using our values in their equation, the estimated modulus of elasticity is around 50% higher on average than the experimental results, but the slope of the two linear regressions is very similar (Figure 10). Silva *et al.* [64] also studied the influence of the MRA incorporation on the modulus of elasticity, through an extensive review with 33 mixes (with diverse cement contents but always higher than the one used in this research). Based on CC’s modulus of elasticity, Silva *et al.*’s loss relative to MRA incorporation ratio was plotted. Silva *et al.*’s experimental regressions show very similar slopes, which could lead to the conclusion that the effect of incorporating increasing MRA content is higher than that of the cement content.

### 3.4. UPV

**Figure 11:**
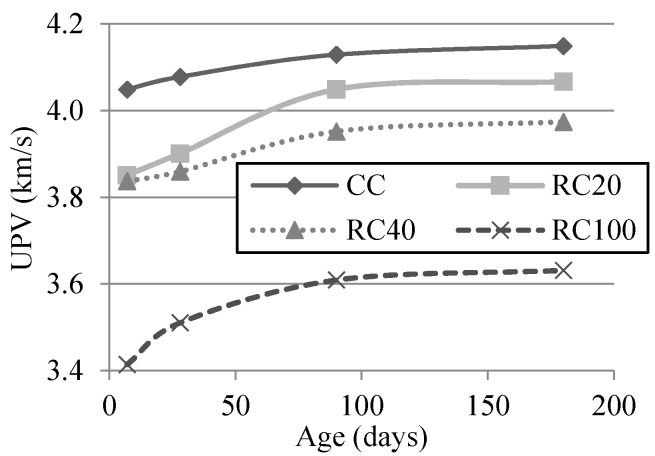
Evolution of the UPV test over the long term.

The evolution of the UPV test over the long term of all mixes is displayed in Figure 11. As expected, the UPV decreased for specimens produced with higher replacement ratio and increased with longer curing times [65,66]. A higher increase over the long term was found in RC100’s UPV than in the rest of the mixes. Kou *et al.* [58] found similar UPV values after 28 days (3.65 km/s) with full coarse replacement of NA with low-grade RA.

**Figure 12:**
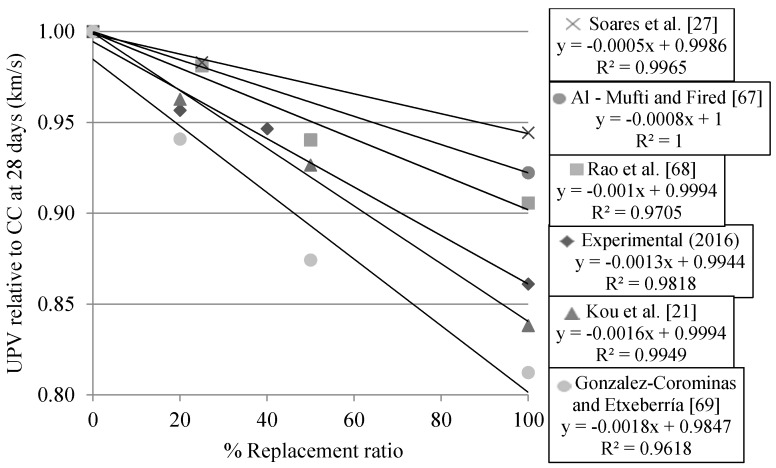
Comparison of UPV relative to that of CC with that of other authors.

UPV values at 28 days relative to MRA’s incorporation ratio compared with those of other authors are presented in Figure 12. Concrete mixes with RCA incorporation [27,67,68] presented less decrease as the replacement level increases than those with MRA incorporation of Kou *et al.* [21], Gonzalez-Corominas and Etxeberría [69] and our results. These trends may be due to the higher quality of the RCA than the MRA, in accordance with Breysse [70], who stated that the main influence on the UPV test results is that of the aggregate and that of other, smaller parameters (e.g., type of cement, cement percentage).

### 3.5. Schmidt Hummer

**Figure 13:**
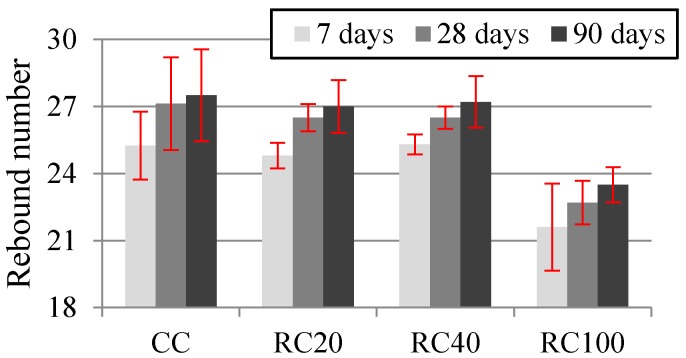
Rebound number at 7, 28 and 90 days.

Figure 13 shows the rebound number of the Schmidt hammer test at 7, 28 and 90 days and the corresponding standard deviation. In spite of the high scatter of this test [71], it was found that the replacement ratio up to 40% had no significant influence on this property, and RC100’s rebound number is lower than that of the rest of the mixes, as for compressive strength.

**Figure 14:**
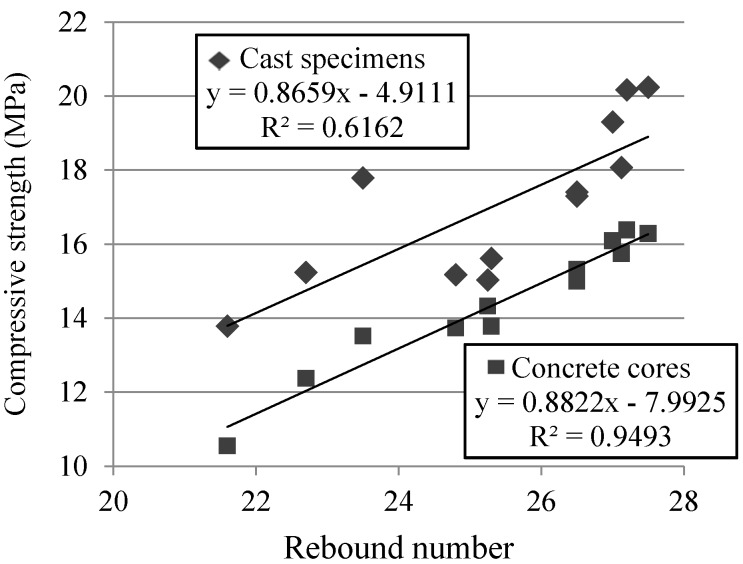
Rebound number at 7, 28 and 90 days.

Figure 14 shows a better correlation between rebound number and compressive strength in concrete cores than in cast specimens. This can be attributed to the fact that cast specimens were cured in wet chamber and that concrete cores have been subjected to the same meteorological conditions.

### 3.6. Physical Properties

Four physical properties related to the durability of concrete, namely saturated surface dry density (SSD density), water penetration under pressure, porosity and water sorptivity, were tested (Table 5). The decrease in SSD density with higher incorporation ratio is due to the lower density of the MRA than the NA. Zaharieva *et al.* [22] obtained, for full MRA incorporation, a similar porosity increase (75%) relative to control concrete and a lower density variation (−3.15%) than in our research. Martinez-Lage *et al.* [13] and Beltrán *et al.* [72] found similar decreases relative to control concrete in SSD density for MRA total replacement: 7.7% and 6.3%, respectively. The values of porosity and water penetration under pressure increased as the incorporation ratio rose. Thomas *et al.* [73] studied the influence of concrete with RCA from CDW with various incorporation ratios on both properties: The variations relative to control concrete with 20% and 100% replacement are lower than those obtained in our research, which could be due to Thomas *et al.*’s study’s having higher cement content and higher-quality RA. Beltrán *et al.* [72] reported 36.3 mm and 70.7 mm in water penetration under pressure of control concrete and full MRA incorporation concrete respectively, which is consistent with our results. Etxeberría *et al.* [24] obtained a sorptivity of 0.74 mm·h^−1/2^ with the same type of cement, similar MRA and 260 kg of cement per m^3^ in concrete produced in a truck mixer, with 50% incorporation ratio, similar to those presented in Table 5. The greater water absorption by capillarity of the mixes with MRA incorporation may have been caused by the higher absorption capacity of the MRA ceramic than that of the NA used in the control mix.

**Table 5:** Physical properties tests conducted in cast specimens.

Physical Properties	SSD-Density		Porosity		Water Penetration		Sorptivity	
Samples	Mg·m^−3^	Variation (%)	%	Variation (%)	mm	Variation (%)	mm·h^−1/2^	Variation (%)
CC	2.174	0.00	11.4	0.00	55	0.00	0.24	0.00
RC20	2.135	−1.79	13.73	20.44	70.3	27.82	0.59	145.8
RC40	2.139	−1.61	13.15	15.35	73.3	33.27	0.61	154.2
RC100	2.038	−6.26	18.05	58.33	91	65.45	0.87	262.5

## 4. Conclusions

This paper presents an upscaled application of recycled concrete slabs to determine the influence of MRA from CDW on the long-term compressive and splitting tensile strengths of concrete cores extracted from the slabs and on the mechanical and durability properties of specimens cast *in situ* with the same conditions as the slabs. Based on the experimental results obtained and their discussion, the conclusions drawn are as follows:

- Compressive strength was similar to that of control concrete by up to 40% incorporation ratio at the same age, in cast specimens and concrete cores.
- Full MRA incorporation concrete cores took one year and 180 days to reach control concrete at 28 days values in compressive and splitting tensile strengths respectively, and high long-term development strength potential was found.
- Comparing the relative strength of the total MRA incorporation compressive strength in cast specimens relative to the control concrete and that of other authors with varying cement content, it was found that the loss of compressive strength is proportionally higher as the strength class increases. The compressive strength in cast specimen reached more than 15 MPa in the total MRA incorporation mix at 28 days, *i.e.*, 15.7% lower than that of the control concrete.
- The ratio between the compressive strength of cast specimens and concrete cores depends on the incorporation ratio, leading to two values; one up to 40% incorporation ratio (1.17) and another for 100% replacements (1.29), in order not to underestimate the latter.
- A reduction in UPV test results associated with MRA incorporation was observed, reaching 16% for full MRA incorporation relative to the control concrete, very similar to that observed in compressive strength.
- The Schmidt hammer test results decreased with MRA incorporation, as expected. A good correlation (0.95) between this parameter and core concrete compressive strength was obtained.
- The use of MRA in concrete has a significant influence on the physical properties related to durability. Nevertheless, this material can be used without steel reinforcement in non-structural applications.

CDW were declared a priority stream waste, and MRA is the most abundant RA produced. In light of the results of this upscaled experimental study, using an MRA that is representative in terms of its ceramic composition by comparison with data from other Spanish authors, the feasibility of the use of concrete with full coarse MRA incorporation and low cement content in non-structural applications, such as bike lanes, gutters, ground slabs, leveling surfaces, subgrades for foundations and similar civil works, is clearly demonstrated.
